# Angiosarcoma secondary to postirradiation and chronic lymphedema: Case reports

**DOI:** 10.1097/MD.0000000000027985

**Published:** 2021-12-03

**Authors:** Jin A. Yoon, Myung Jun Shin, Yong Beom Shin, Byeong Ju Lee, Kyung Un Choi, Joo Hyoung Kim

**Affiliations:** a Department of Rehabilitation Medicine, Pusan National University School of Medicine and Biomedical Research Institute, Pusan National University Hospital, Busan, Republic of Korea; b Department of Pathology, School of Medicine, Pusan National University, Yangsan, Republic of Korea; c Department of Plastic and Reconstructive Surgery, Busan Cancer Center, Pusan National University Hospital and Biomedical Research Institute, Pusan National University Hospital, Busan, Republic of Korea.

**Keywords:** angiosarcoma, cancer, lymphedema

## Abstract

**Introduction:**

Angiosarcoma secondary to post-irradiation and lymphedema is rare, but it is aggressive with a poor prognosis. It is essential to understand these patients’ clinical features and distinguish them from benign diseases or other malignant tumors.

**Patient concerns:**

Three patients who had radiotherapy for cancer treatment and chronic lymphedema admitted to the hospital with specific skin lesions at upper or lower extremities.

**Diagnosis:**

Excisional biopsies revealed prominent, highly atypical cells with a vasoformative area, composed of atypical, large epithelioid cells with vesicular nuclei, prominent nucleoli, and mitoses. Immunohistochemistry revealed diffuse expression of endothelial cell markers suggestive of angiosarcoma.

**Interventions:**

One patient had shoulder disarticulation with wide excision with adjuvant radiotherapy and chemotherapy and other 2 discontinued the treatment.

**Outcomes:**

After the treatment, one patient was transferred to rehabilitation department for shoulder disarticulation prosthesis fitting without recurrence sign for 1 year. Two patient refused further treatment and was lost to follow-up.

**Conclusion:**

In cases of patients with irratiation and chronic lymphedema, clinical findings suggestive of angiosarcoma, biopsy and imaging studies should be performed as soon as possible.

## 1 Introduction

Angiosarcoma is a rare and aggressive vascular neoplasm of endothelial origin with a poor prognosis. The median survival time is 2.5 years, with 20% to 40% having distant metastasis at diagnosis.^[1]^ It is associated with radiation treatment and chronic lymphedema following cancer treatment, but some cases are idiopathic.^[2,3]^ Cutaneous angiosarcoma that presents as moderate- or high-grade lesions at diagnosis is common. Thus, early diagnosis and prompt surgery are essential to prolonging survival.^[4]^ The clinical manifestation of angiosarcoma should be identified by dermatologists and oncologists. We report three angiosarcoma cases to demonstrate its clinical aspects and emphasize the possibility of cutaneous angiosarcoma in patients after cancer treatment. The patients have provided informed consent for the publication of the case report. This study was approved by our institutional review board, and the requirement for written consent was waived (IRB No. H-2009–026–095).

## 2 Case reports

### 2.1 Case 1

**Figure 1:**
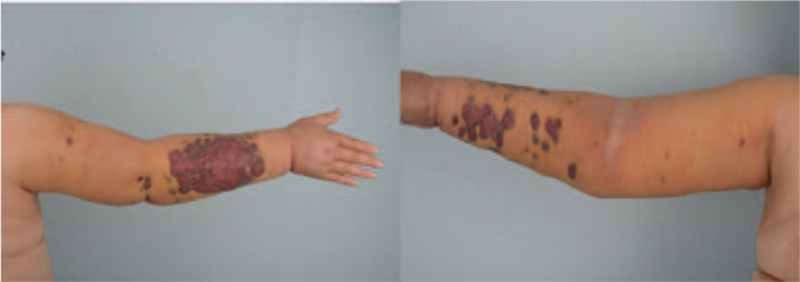
Clinical presentation of purplish, lobulated macules on the right forearm extending to the upper arm in case 1.

**Figure 2:**
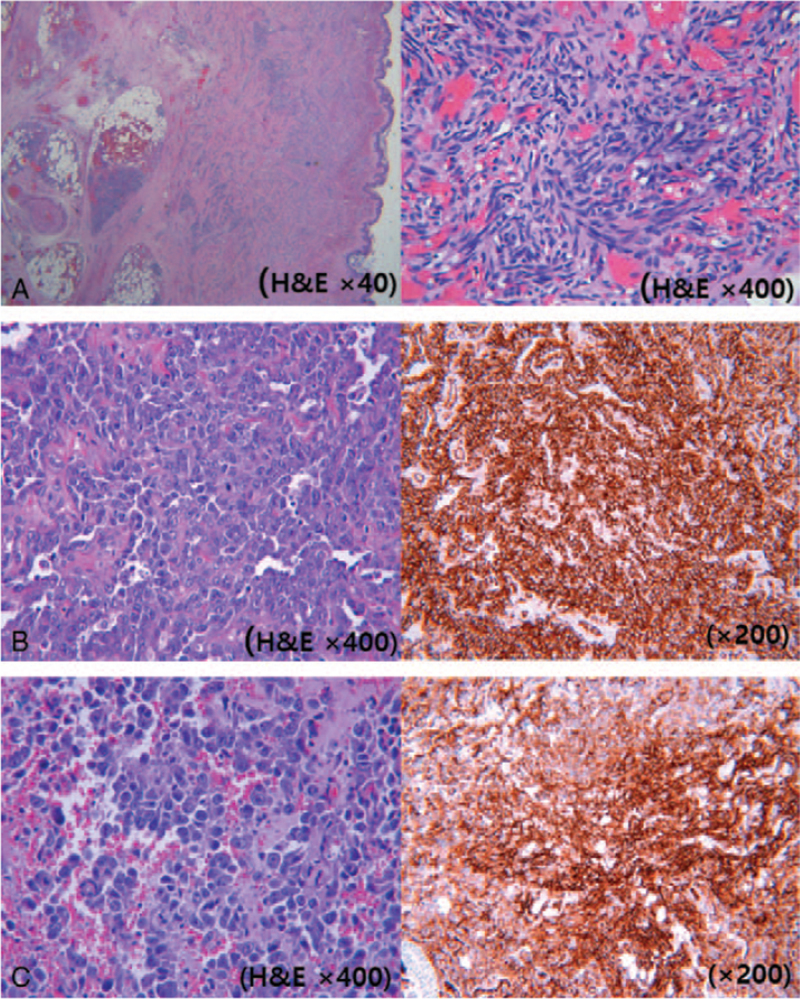
Histologic and immunohistochemical features of the patients. A. The skin shows highly atypical cells with vasoformative areas (H&E, ×40) and large epithelioid cells with pleomorphic nuclei and prominent nucleoli (H&E, ×400) in case 1. B and C. The tumor is composed of large, atypical epithelioid cells with vesicular nuclei, prominent nucleoli, and mitoses (H&E, ×400), and CD31 immunostaining shows diffuse positivity for tumor cells (×200) in cases 2 and 3.

A 62-year-old woman who underwent quadrantectomy with a latissimus dorsi muscle flap, chemotherapy, and radiotherapy for right breast cancer 21 years ago was admitted to the hospital. She had extensive chronic lymphedema of the right arm for 10 years and painless multiple purplish, lobulated macules on the forearm 2 months ago. Although there was no local tenderness or increased temperature, the purpuric skin lesions spread to the proximal arm, and the forearm lesions rapidly enlarged over the past 2 weeks (Fig. 1). There were no clinical or laboratory findings suggestive of cellulitis. First, the patient was referred for magnetic resonance imaging (MRI) of the right forearm to evaluate the underlying lesion associated with her skin transformation. Axial tau inversion recovery and T1-weighted images of the right arm showed extensive subcutaneous edema and skin thickening suggestive of chronic lymphedema. There was no evidence of intrathoracic or distant metastasis on chest computed tomography and positron emission tomography–computed tomography (PET-CT) (F18-fluorodeoxyglucose [FDG]). There were extensive subcutaneous edema and skin thickening with diffuse uptake in the forearm (SUVmax, 3.0) on PET-CT. Excisional biopsy revealed prominent, highly atypical cells with a vasoformative area composed of atypical, large epithelioid cells with vesicular nuclei, prominent nucleoli, and mitoses (Fig. 2-A). Immunohistochemistry revealed a diffuse expression of endothelial cell markers CD31, CD34, and ERG. It was focal positive for D2-40, and lymphatic markers suggested spindle cell angiosarcoma (pT4N0, G1) and Stewart–Treves syndrome type I (strongly positive for CD31 and partially positive for D2-40).^[6–8]^ The expression index of the Ki-67 value was 30%. The tumor was located in the dermis. Shoulder disarticulation with wide excision of the shoulder mass and reconstruction with local flap advancement was performed. Adjuvant Rtx (75 Gy/#35) and taxol Cx. C3 was performed shortly thereafter. She was transferred to the rehabilitation department for shoulder disarticulation prosthesis fitting and received physical therapy without recurrence for 1 year.

### 2.2 Case 2

**Figure 3:**
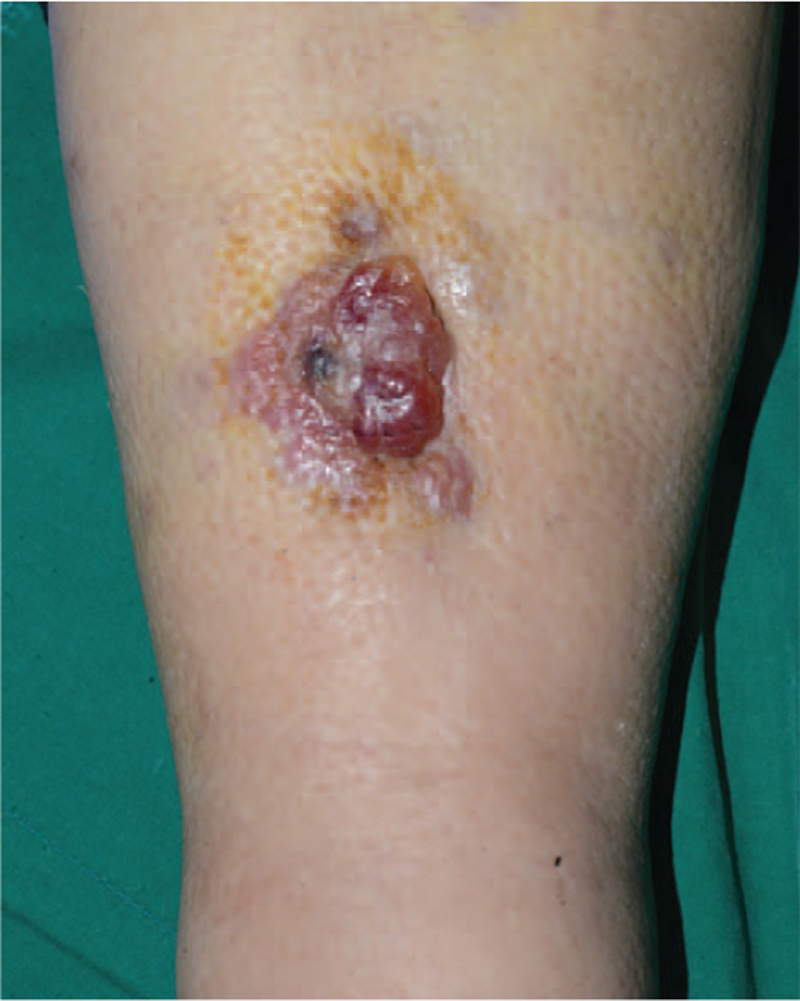
The erythematous and granulomatous plaque measuring 1.6 × 1.0 cm at the right shin in case 2.

A 77-year-old woman with a history of hysterectomy and radiotherapy for cervical cancer 25 years ago visited our hospital for right leg edema lasting 8 years. The patient was admitted for an unhealed soft-tissue defect accompanied by exudate from the right lower extremity. She received medical treatment for lymphedema on the right leg at the rehabilitation clinic. She then visited the hospital for evaluation since a shin wound with prolonged exudate from exercise did not heal for 2 months. She had no history of trauma or falls. The lesion was an erythematous and granulomatous plaque measuring 1.6 × 1.0 cm at the time of her visit. The wound's healing was delayed by maceration due to lymphorrhea in the intercurrent wound, initially caused by lymphedema. Progress was observed while applying hydrofiber and foam dressing for exudate control. Even after continuous follow-up for 3 months, the lesion did not improve and demonstrated signs of spreading (Fig. 3). MRI and PET-CT documented the involvement of the intermuscular fascia and bone marrow. Debridement of the soft-tissue defect was performed. Histologic analysis revealed large, highly atypical epithelioid cells with vesicular nuclei, prominent nucleoli, and mitoses, extending to the dermal and subcutaneous regions. The tumor cells stained positive for CD31 and CD34 and negative for SMA and EMA (Fig. 2-B). The pathologic diagnosis was compatible with angiosarcoma. The patient refused further treatment and was discharged from the hospital.

### 2.3 Case 3

**Figure 4:**
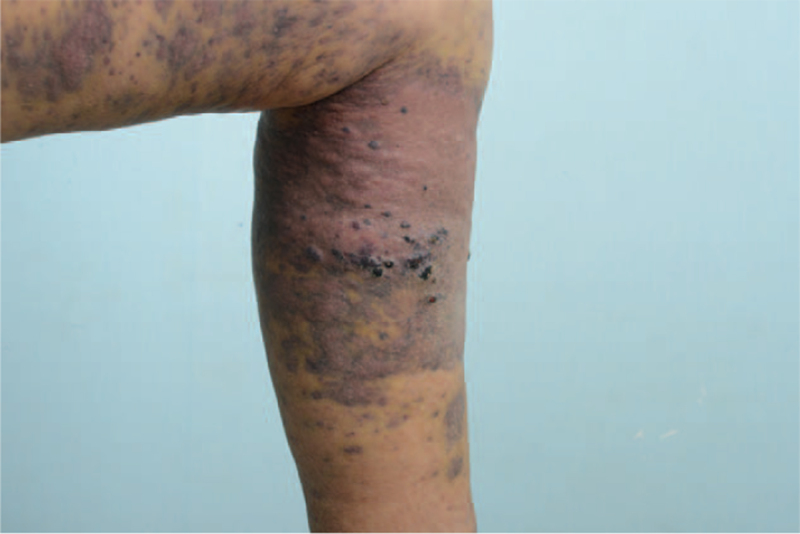
The erythematous, purplish, lobulated plaque on the left lower leg and thigh in case 3.

A 76-year-old woman was referred to our clinic to assess an erythematous, purplish, lobulated plaque on her left lower leg and thigh lasting for 5 months (Fig. 4). The patient underwent Moh's operation and local radiotherapy (50.4 Gy #28) for squamous cell carcinoma of the left thigh and inguinal LN metastasis (pT2NiMo) 12 years ago. Although the patient had lymphedema of the left lower extremity for 6 years with recurrent cellulitis, she was afebrile and had no leukocytosis at the time of visit. Therefore, an excisional biopsy was performed to establish the diagnosis. The pathology revealed highly atypical cells with vasoformative areas, which were compatible with angiosarcoma. Immunohistochemical findings showed diffuse expression of CD31 and CD34 (focal) (Fig. 2-C). It tested negative for EMA. These findings were suggestive of epithelioid angiosarcoma. MRI findings showed diffuse skin thickening in the lower leg, predominantly in the pretibial area from the proximal to the mid diaphysis level and medial proximal lower leg with diffuse enhancement. LN metastasis in the left inguinal LN was suspected on PET-CT. We planned to proceed with chemotherapy (taxol weakly C8). During a total of three chemotherapy sessions, the lesion was accompanied by recurrent cellulitis, and treatment was discontinued thereafter. The patient refused further treatment and was lost to follow-up.

## 3 Discussion

Angiosarcoma secondary to postirradiation and lymphedema is rare, but it is aggressive with a poor prognosis.^[9]^ The frequency is estimated at approximately 0.45%, and the appearance of the lesion occurs within 10 years on average, ranging from 1 to 26 years. The risk of recurrence at 5 years is approximately 84%. The poor prognostic factors include large tumor size, depth of invasion greater than 3 mm, high mitotic rate, positive surgical margins, and metastasis.^[5]^ The exact mechanism and role of chronic lymphedema and angiosarcoma are unknown. In chronic lymphedema, lymphostasis may produce local immunodeficiency, inducing vascular oncogenesis. Radiation therapy may cause lymph node sclerosis, leading to local lymphedema and, thus, an immunocompromised state.^[10]^ Its prevalence has decreased after substantial improvement in surgical radiation therapy. Due to this disease's rarity and poor prognosis, even experienced doctors may not have encountered this tumor previously.

It is essential to understand these patients’ clinical features and distinguish them from benign diseases or other malignant tumors. Multiple spreading reddish-blue or purplish macules, as seen in cases 1 and 3, are known symptoms. These are followed by the development of palpable subcutaneous nodule tissue infiltration and eschar with recurrent bleeding in patients with chronic lymphedema.

Establishing differential diagnoses for angiosarcoma is also essential to distinguish it from benign diseases, such as chronic nonhealing venous ulcers and other malignant vascular diseases. In case 2, the lesion occurred after the fall, and the treatment was performed due to maceration after lymphedema. However, a biopsy was performed because it did not heal. Additionally, Kaposi sarcoma exhibits clinical features similar to angiosarcoma and can occur after lymphedema. Therefore, immunohistochemical testing is essential, and the presence of human herpesvirus 8 distinguishes them.^[11]^

Angiosarcoma is difficult to diagnose as it mimics other benign tumors on MRI or PET-CT, often leading to delayed diagnosis. In addition, reports describing the MRI findings of the early stages of angiosarcoma are rare. In our patients, the MRI signal intensity was normal. In addition, only a mild FDG uptake was shown at the origin site, which is inconsistent with findings known for aggravating vascular tumors in case 1.^[12]^ Therefore, in cases of new dermatologic findings in chronic lymphedema, the possibility of angiosarcoma should not be excluded, and additional biopsies must be considered to confirm the histopathologic findings.

The histologic features of angiosarcoma include abnormal, atypical large epithelioid cells with pleomorphic nuclei and prominent nucleoli. The endothelial cells are round or oval, with prominent mitosis.^[13]^ CD31 (cat. #RMPD057), CD34 (cat. #RMPD058), and ERG (cat. #RMPD034) are markers for vascular differentiation and endothelial cells. CD31 expression, used together with CD34 and ERG, is the gold standard for identifying endothelial differentiation in angiosarcoma.^[6]^

In addition, angiosarcoma and lymphangiosarcoma (LAS) are often used interchangeably. D2-40 is an available marker for lymphatic endothelium, and its sensitivity for LAS was reportedly excellent. In patients with mixed immunophenotype, tumor cells may demonstrate partial differentiation along the lymphatic endothelial lineage. In our patient, strong positivity for CD31 and weak positivity for D2-40 were more suggestive of Stewart-Treves syndrome type I than of LAS or mixed LAS.^[8,14]^

The treatment of angiosarcoma involves surgery and chemotherapy/radiotherapy. However, there are no evidence-based recommendations for specific angiosarcoma subtypes yet.^[15]^ Despite the low mitotic rate and ambiguous FDG uptake that was shown on PET-CT, shoulder disarticulation with additional wide excision at the stump site was performed for case 1 because the extensive skin lesion crossed multiple joints and had a depth of invasion of more than 3 mm.^[16]^ Despite aggressive and multimodal treatment, the overall prognosis was poor, with a median survival of 2.5 years after diagnosis.^[15]^ Therefore, early and accurate detection is vital. These cases showed that chronic lymphedema could evolve into a fatal entity despite its harmless nature. Therefore, for patients with chronic lymphedema, especially those who had surgery in the past 5 years, regular follow-up is essential. In cases with dermatologic findings suggestive of angiosarcoma, biopsy and imaging studies should be performed as soon as possible.

### 3.1 Patient consent statement

The patients have provided informed consent for the publication of this case.

## Author contributions

**Conceptualization:** Jin A Yoon, Joo Hyoung Kim, Myung Jun Shin.

**Data curation:** Joo Hyoung Kim, Kyung Un Choi.

**Supervision:** Byeong Ju Lee.

**Writing – original draft:** Jin A Yoon, Joo Hyoung Kim.

**Writing – review & editing:** Joo Hyoung Kim, Myung Jun Shin, Yong Beom Shin, Byeong Ju Lee.
